# Re-esterified Palm Oils, Compared to Native Palm Oil, do not Alter Fat Absorption, Postprandial Lipemia or Growth Performance in Broiler Chicks

**DOI:** 10.1007/s11745-014-3920-9

**Published:** 2014-06-17

**Authors:** E. Vilarrasa, A. Tres, L. Bayés-García, T. Parella, E. Esteve-Garcia, A. C. Barroeta

**Affiliations:** 1 Animal Nutrition and Welfare Service (SNiBA), Department of Animal and Food Science, Facultat de Veterinària, Universitat Autònoma de Barcelona, 08193 Bellaterra, Barcelona Spain; 2 Nutrition and Food Science Department, XaRTA, INSA, Facultat de Farmàcia, Universitat de Barcelona, Joan XXIII s/n, 08028 Barcelona, Spain; 3 Departament de Cristal·lografia, Mineralogia i Dipòsits Minerals, Facultat de Geologia, Universitat de Barcelona, Martí i Franquès s/n, 08028 Barcelona, Spain; 4 Servei de Ressonància Magnètica Nuclear, Facultat de Ciències, Universitat Autònoma de Barcelona, 08193 Bellaterra, Barcelona Spain; 5 Monogastric Nutrition, Institut de Recerca i Tecnologia Agroalimentàries (IRTA), Ctra. de Reus-El Morell Km 4.5, 43120 Constantí, Tarragona Spain

**Keywords:** *sn*-2 position, Monoacylglycerol, Diacylglycerol, Fatty acid apparent absorption, Postprandial lipemia, Solid fat index

## Abstract

Re-esterified palm oils are obtained from the chemical esterification of palm acid oils (rich in free fatty acids) with glycerol, both economically interesting by-products from oil refining and biodiesel industries, respectively. Thus, re-esterified palm oils could be an economically interesting alternative to native palm oil in broiler chick diets. However, because they may have different physicochemical properties than have their corresponding native oil, we assessed the effect of fatty acid (FA) positional distribution within acylglycerol molecules and the effect of acylglycerol composition on FA apparent absorption, and their possible consequences on the evolution of postprandial lipemia and growth performance in broiler chicks. Seventy-two 1-day-old female broiler chicks were randomly distributed into 18 cages. The three treatments used were the result of a basal diet supplemented with 6 wt% of native palm oil (N-TAG), re-esterified palm oil (E-TAG), or re-esterified palm oil high in mono- and diacylglycerols (E-MDAG). Chemical esterification raised the fraction of palmitic acid at the *sn*-2 position from 9.63 mol% in N-TAG oil to 17.9 mol% in E-TAG oil. Furthermore, E-MDAG oil presented a high proportion of mono- (23.1 wt%) and diacylglycerols (51.2 wt%), with FA mainly located at the *sn*-1,3 positions, which resulted in a lower gross-energy content and an increased solid-fat index at the chicken’s body temperature. However, re-esterified palm oils did not alter fat absorption, postprandial lipemia, or growth performance, compared to native palm oil, so they can be used as alternative fat sources in broiler chick diets.

## Introduction

Among the ingredients used in the formulation of animal diets, fats and oils are the most concentrated sources of energy, but also those with the most variable nutritive value [1]. The biological role of the chain length and the degree of unsaturation of fatty acids (FA) is well known [2]. However, evidence is accumulating that the intramolecular structure of dietary fats is also of importance because it can affect their rates of digestion and absorption, and also its subsequent metabolism. Both the number of FA bound to glycerol molecules and the stereospecific position of FA within acylglycerol molecules play an important role in fat absorption. Tri- (TAG) and diacylglycerols (DAG) are too-large molecules and cannot be absorbed intact in the small intestine. Thus, before absorption, they must be broken down by pancreatic lipase to monoacylglycerols (MAG) and free fatty acids (FFA). Pancreatic lipase preferentially hydrolyzes the FA in the *sn*-1,3 positions of the acylglycerol molecules [3]. Consequently, FA in the *sn*-2 position predominantly remain in this location and are directly absorbed as 2-MAG. 2-MAG are easily absorbed regardless of their constituent FA, because their amphiphilic properties facilitate their incorporation into mixed micelles [4, 5]. However, the absorption of FFA varies greatly depending on their chemical structure. Mono- (MUFA) and polyunsaturated fatty acids (PUFA) are well absorbed, but long-chain saturated fatty acids (SFA) are poorly absorbed because they have high hydrophobicity, high melting points above body temperature, and a great ability to form insoluble soaps with divalent cations in the gut [6]. Therefore, stearic and palmitic acids are better absorbed if they are situated in the *sn*-2 position of the acylglycerol molecules than in the *sn*-1,3 positions [7–12].

Due to market availability and competitive price [13], native palm oil is a high-energy ingredient to be considered in animal nutrition. However, its high content of SFA, predominately esterified at the TAG *sn*-1,3 positions, compromises its absorption and, therefore, its nutritive value, especially at early ages [14, 15]. Re-esterified palm oils are obtained from the chemical esterification of palm acid oils (rich in FFA) with glycerol, both economically interesting by-products from oil refining and biodiesel industries, respectively. This process, in addition to giving added value to acid oils, which have a known low apparent metabolizable energy due to its high FFA content [15, 16], results in the development of new fat sources, which could be an economically interesting alternative to native palm oil. It was hypothesized that the different acylglycerol composition and FA positional distribution within acylglycerol molecules of re-esterified palm oils may enhance the SFA apparent absorption, and thus increase their nutritive value.

However, to-date, and to the knowledge of the authors, there are no reports in the literature using this kind of re-esterified palm oils. Because re-esterified oils may have different physicochemical properties than have their corresponding native oil, we assessed the effect of FA positional distribution within acylglycerol molecules, and the effect of acylglycerol composition on FA apparent absorption, and their possible consequences on the evolution of postprandial lipemia and growth performance, in order to determine if re-esterified palm oils can be used as alternative fat sources to native palm oil in broiler chick diets.

## Materials and Methods

### Experimental Fats

Experimental fats were supplied by SILO S.p.a. (Florence, Italy). Re-esterified palm oils were produced taking, as raw materials, palm acid oil (a by-product obtained from the refining process of crude palm oil, with a high FFA content) and glycerol (a by-product obtained from the methylation process applied for biodiesel production), which were processed in a reactor for 4–6 h, under high vacuum conditions (1–3 mm Hg), at temperatures between 190–250 °C, and without chemical catalysts. According to the stoichiometric proportion of FFA and glycerol, fats with the same FA profile, but with a different FA positional distribution, and TAG, DAG and MAG proportions were obtained (Table 1).

**Table 1:** Chemical analyses of the experimental fats

Item	N-TAG oil	E-TAG oil	E-MDAG oil
Moisture (wt%)	0.03	0.01	0.07
Impurities (wt%)	<0.50	<0.50	<0.50
Unsaponifiable matter (wt%)	1.44	1.03	1.38
Fatty acid composition and distribution (wt%)			
C16:0			
Total	43.5	44.5	46.9
*sn*-2 %^a^	9.63	17.9	14.5
C18:0			
Total	4.60	4.51	4.76
*sn*-2 %^a^	11.6	18.4	19.5
C18:1n-9			
Total	38.6	39.3	36.1
*sn*-2 %^a^	38.5	41.7	20.1
C18:2n-6			
Total	10.1	9.19	8.40
*sn*-2 %^a^	42.8	49.3	19.9
Minor fatty acids	3.25	2.56	3.79
SFA			
Total	49.5	50.2	53.9
*sn*-2 %^a^	10.2	18.3	14.6
MUFA			
Total	39.8	40.5	37.6
*sn*-2 %^a^	37.9	41.3	20.1
PUFA			
Total	10.7	9.33	8.58
*sn*-2 %^a^	42.4	49.3	19.9
Acylglycerol composition (wt%)			
TAG	66.6	86.2	25.6
DAG			
Total	19.9	12.1	51.2
1(3),2-DAG %^b^	16.7	13.6	31.5
MAG			
Total	2.08	0.40	23.1
2-MAG %^c^	12.5	50.0	6.45
FFA	11.4	1.32	0.00
Glycerol-to-fatty acid ratio^d^ (mol/mol)	0.34	0.35	0.56
Gross energy (kcal/kg)	9,307	9,298	8,947

Native palm oil (N-TAG oil), re-esterified palm oil (E-TAG oil), and re-esterified palm oil high in MAG and DAG (E-MDAG oil)

*SFA* saturated fatty acids, *MUFA* monounsaturated fatty acids, *PUFA* polyunsaturated fatty acids, *TAG* triacylglycerols, *DAG* diacylglycerols, *MAG* monoacylglycerols, *FFA* free fatty acids

^a^The proportion of a particular FA that is located at the acylglycerol *sn*-2 position (*sn*-2 %) was calculated as follows: *sn*-2 % = (*sn*-2/Total) × *a* × 100, where *sn*-2 is the FA composition at the *sn*-2 position, Total is the total FA composition in the original fat, and *a* is the ratio between the moles of FA located at the *sn*-2 position and the moles of total FA. *a* was 0.24, 0.30 and 0.17 for N-TAG, E-TAG and E-MDAG oils, respectively

^b^The proportion of 1(3),2-DAG relative to total DAG

^c^The proportion of 2-MAG relative to total MAG

^d^Estimated calculation based on the values of the acylglycerol composition

Each oil sample was analyzed in triplicate. Moisture (Method 926.12 of the AOAC [17]), impurities (ISO 663:2007), and unsaponifiable matter (Method 933.08 of the AOAC [17]) (MIU) content was determined as a quality control.

**Fig. 1:**
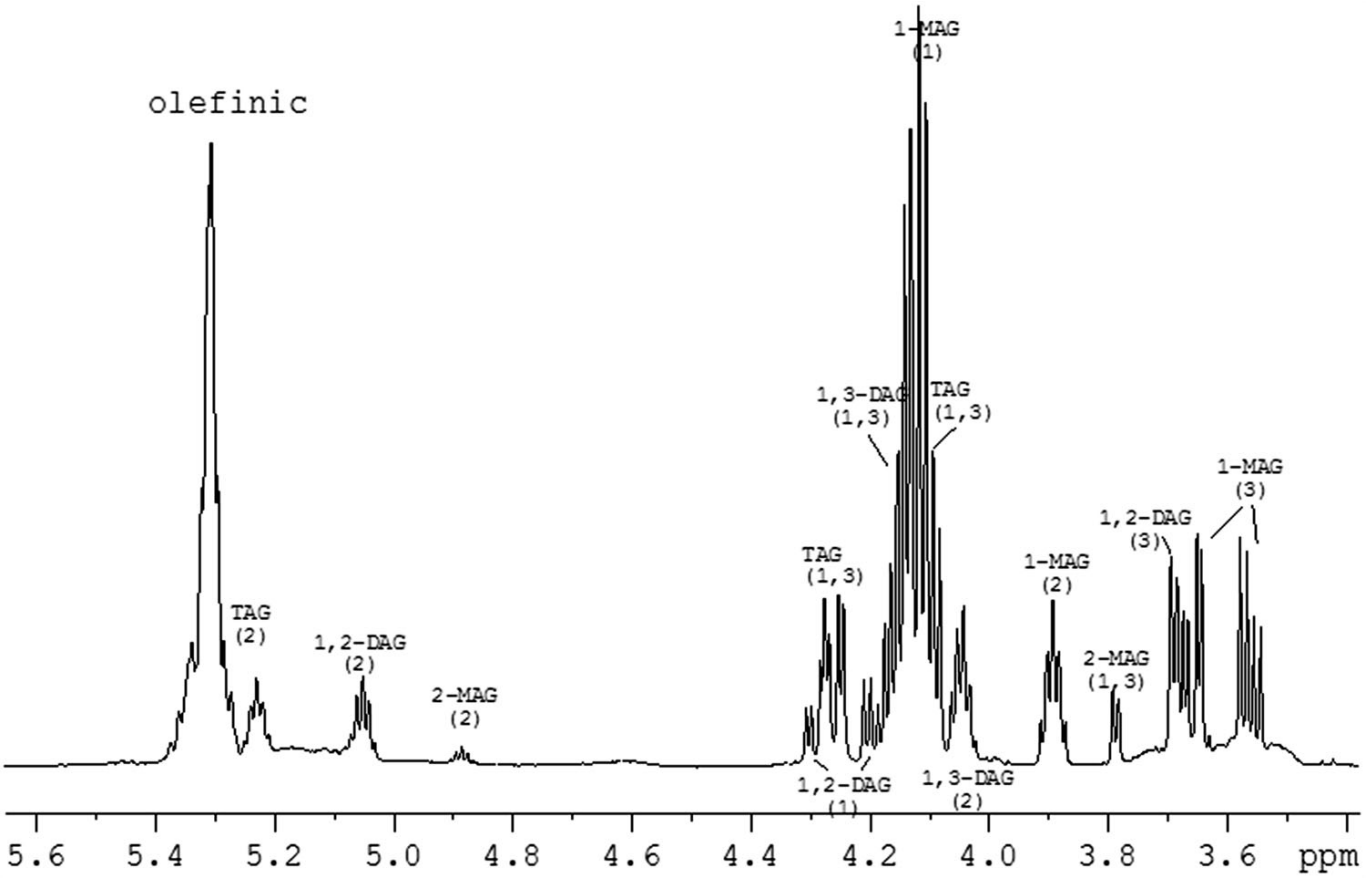
Expanded area of the ^1^H-NMR spectrum of a fat sample, with the ^1^H chemical shift assignment of the glycerol signals corresponding to the differently substituted glycerol units

The acylglycerol composition of experimental fats was analyzed according to the ISO 18395:2005, in which TAG, DAG, MAG, and FFA are separated according to their molecular size. Briefly, a solution of approximately 10 mg of oil/mL of tetrahydrofuran was injected into an Agilent 1100 series HPLC chromatograph (Agilent Technologies; Santa Clara, CA) equipped with a refractive index detector and two Styragel columns (Styragel HR 1 and Styragel HR 0.5) of 30 cm × 0.78 cm i.d., filled with a spherical styrene divinylbenzene copolymer of 5 μm particle size (Water Associates; Milford, MA) connected in series. The mobile phase consisted of tetrahydrofuran. The acylglycerol molecules were quantified by internal normalization. Moreover, given the potential importance of different positional isomers of MAG and DAG molecules in the digestion and absorption processes, we also analyzed the experimental fats by high-resolution ^1^H nuclear magnetic resonance (NMR) spectroscopy. Thus, 2-MAG were distinguished from 1(3)-MAG, and 1(3),2-DAG from 1,3-DAG species by area integration of the individual resonances corresponding to the central CH at the *sn*-2 position in each type of compound. These species can be detected in the area covering 5.3–3.8 ppm, clearly differentiating the H2 protons belonging to 1(3),2-DAG (5.05 ppm), 1,3-DAG (4.03 ppm), 2-MAG (4.88 ppm) and 1(3)-MAG (3.89 ppm) derivatives (Fig. 1). The degree of unsaturation and the chain length of FA do not influence the chemical shift values [18]. Briefly, oil samples (about 6 mg) were dissolved in deuterated chloroform and placed into a 5-mm-diameter NMR tubes. Conventional one-dimensional ^1^H-NMR spectra were collected under routine conditions on a Bruker 600 MHz spectrometer (Bruker; Billerica, MA) equipped with a triple-channel TXI probe. All experiments were recorded at 298 K, using a recycle delay of 3 s and four scans per sample. After Fourier transformation and base-line correction, the areas of the selected H2 proton signals of the spectrum were quantified by area integration.

The total FA composition of experimental fats was determined by gas chromatography, according to the methylation method described by Guardiola et al. [19]. Briefly, 50 mg of oil were methylated with sodium methoxide (0.5 N), followed by boron trifluoride (20 wt% in methanol) and FA methyl esters were extracted with *n*-hexane. Subsequently, FA methyl esters were analyzed using an Agilent 4890D gas chromatograph (Agilent Technologies; Santa Clara, CA) equipped with a flame ionization detector and a polar capillary column (SP-2380, 60 m × 0.25 mm i.d., 0.2 μm from Supelco; Bellefonte, PA). Helium was used as the carrier gas. FA methyl esters were identified by matching their retention times with those of their relative standards (Supelco 37 component FAME Mix, Sigma-Aldrich Co.; St. Louis, MO) and quantified by internal normalization.

The FA composition at the *sn*-2 position of the acylglycerol molecules was determined by the EU official method (Commission Regulation (EEC) No. 2568/91—Annex VII). Briefly, the original fat was hydrolyzed by pancreatic lipase (EC 3.1.1.3 from porcine pancreas Type II, Sigma-Aldrich Co.; St. Louis, MO) to selectively cleave the ester bonds at the *sn*-1,3 positions. 2-MAG were isolated by thin-layer chromatography using silica gel plates (Merck; Darmstadt, Germany) impregnated in boric acid (5 wt% in methanol). The 2-monoolein and 1-monoolein standards (Sigma-Aldrich Co.; St. Louis, MO) were spotted for identifying the 2-MAG zone spot. The developing solvent was a mixture of chloroform/acetone (90:10, by vol). The zone spot was visualized under UV light after being sprayed with 0.2 wt% of 2,7-dichlorofluorescein in methanol. Then, 2-MAG were scraped, and the FA composition of 2-MAG was determined as described above. Finally, to assess the distribution of each FA within the three positions of the acylglycerol molecules, a modification of the formula suggested by Mattson [20] was used. These authors calculated the proportion of each FA that is located at the *sn*-2 position of the acylglycerol molecules (*sn*-2 %) applying the following formula:

1 $$sn{ - }2{\mkern 1mu}\; \% = \left( {sn{ - }2/{\text{Total}}} \right) \times a \times 100$$

where *sn*-2 is the FA composition at the *sn*-2 position (converted to mol%), Total is the total FA composition in the original fat (converted to mol%), and *a* is the ratio between the moles of FA located at the *sn*-2 position and the moles of total FA. Thus, in the original formula [20], *a* was equal to 0.33, since it was designed for native oils that are mainly constituted by TAG. In our study, however, experimental fats were a mixture of TAG, DAG, MAG, and FFA. For this reason, *a* was calculated from the acylglycerol composition of the fat, and the average molecular weight (according to the total FA composition of the fat) and the glycerol-to-FA ratio for each molecular species. These calculations were also used to obtain an estimation of the global glycerol-to-FA ratio of our experimental fats (Table 1).

Additionally, because changes in both FA positional distribution and acylglycerol composition may cause differences in the physical properties of the fat, the melting behavior of the experimental fats was studied by differential scanning calorimetry (DSC; Perkin-Elmer Diamond Calorimeter; Waltham, MA). Briefly, oil samples (about 6 mg) were weighed into 50-μl aluminium pans, and covers were sealed into place. An empty pan was used as a reference. Samples were cooled and heated at 2 °C min^−1^ between −60 and 60 °C. Thermograms were analyzed to obtain the total and partial melting enthalpies; assuming that the total melting enthalpy of a fat is the total energy required to convert the substance from a solid state to a complete melt. Then, the solid fat index was calculated for each 5 °C interval. The result of these calculations is a graphic indication of the loss of solids due to the melting process.

Finally, combustion energies of the experimental fats were measured by an adiabatic bomb calorimeter (IKA-Kalorimeter system C4000; Staufen, Germany).

### Animals and Diets

The trial was performed at the animal experimental facilities of the Servei de Granges i Camps Experimentals (Universitat Autònoma de Barcelona; Bellaterra, Barcelona, Spain). The experimental procedure received prior approval from the Animal Protocol Review Committee of the same institution. All animal housing and husbandry conformed to the European Union Guidelines (2010/63/EU).

A total of 72 one-day-old female broiler chickens of the Ross 308 strain were obtained from a commercial hatchery (Pondex SAU; Juneda, Lleida, Spain) where birds with extreme weights were discarded. On arrival, chicks were wing-banded, weighed (initial body weight, 46.5 ± 3.03 g) and randomly assigned to one of the three dietary treatments, with four chicks per cage and six cages per treatment. Birds were housed in wire-floor cages with excreta collection trays. Throughout the study, feed and water were supplied ad libitum, and animals were raised under controlled conditions of light and temperature, as recommended by the breeder.

The birds received a starter feed (in mash form) until day 14. The wheat- and soybean-meal-based diet was formulated to meet or exceed FEDNA requirements [21] and to minimize basal fat levels. The three dietary treatments were the result of including 6 wt% of one of the following experimental fats to the basal diet: native palm oil (N-TAG), re-esterified palm oil (E-TAG), or re-esterified palm oil high in MAG and DAG (E-MDAG). The composition of experimental diets is presented in Table 2. The manufacturing of the experimental diets was carried out at the experimental station of IRTA Mas de Bover (Constantí, Tarragona, Spain).

**Table 2:** Ingredient composition of the experimental diets

Ingredients (wt%)	
Wheat	51.4
Soybean meal 48 %	38.6
Experimental fats^a^	6.00
Dicalcium phosphate	1.69
Calcium carbonate	1.30
Sodium chloride	0.40
Vitamin and mineral pre-mix^b^	0.30
DL-Methionine	0.23
l-Lysine	0.07
Enzyme supplement^c^	0.05
Antioxidant^d^	0.02

^a^Native palm oil (N-TAG), re-esterified palm oil (E-TAG), or re-esterified palm oil high in mono- and diacylglycerols (E-MDAG)

^b^Provides per kg of feed: vitamin A (from retinol) 13,500 IU; vitamin D_3_ (from cholecalciferol) 4,800 IU; vitamin E (from alpha–tocopherol) 49.5 IU; vitamin B_1_, 3 mg; vitamin B_2_, 9 mg; vitamin B_6_, 4.5 mg; vitamin B_12_, 16.5 µg; vitamin K_3_, 3 mg; calcium pantothenate, 16.5 mg; nicotinic acid, 51 mg; folic acid, 1.8 mg; biotin, 30 µg; Fe (from FeSO_4_·7H_2_O) 54 mg; I (from Ca(I_2_O_3_)_2_) 1.2 mg; Co (from 2CoCO_3_·3Co(OH)_2_·H_2_O) 0.6 mg; Cu (from CuSO_4_·5H_2_O) 12 mg; Mn (from MnO) 90 mg; Zn (from ZnO) 66 mg; Se (from Na_2_SeO_3_) 0.18 mg; Mo (from (NH_4_)_6_Mo_7_O_24_) 1.2 mg

^c^Provides per kg of feed: β-glucanase 350 IU; xylanase 1,125 IU

^d^Ethoxyquin 66 %

Analytical determinations of feeds were performed according to the methods of AOAC [17]: Dry matter (Method 934.01), ash (Method 942.05), crude protein (Method 968.06), crude fat (Method 2003.05), and crude fiber (Method 962.09). Gross energy was determined as described previously, and FA content was analyzed following the method of Sukhija and Palmquist [22], which consists of a direct transesterification in which lipid extraction and FA methylation are achieved in only one step. Briefly, samples (about 100 mg) were incubated with methanolic chloride, and a known amount of nonadecanoic acid (C19:0, Sigma-Aldrich Chemical Co.; St. Louis, MO) was added as an internal standard. Then, the FA methyl esters were extracted with toluene and submitted to gas chromatography (Agilent 6890 gas chromatograph, equipped with a flame ionization detector, and a polar capillary column [DB23, 60 m × 0.32 mm i.d., 0.25 μm] from Agilent Technologies; Santa Clara, CA). Helium was used as the carrier gas. Peak areas were integrated and converted to concentration by comparison with the internal standard-peak area, as follows:

2 $${\text{FA}} = \left( {\text{area FA/area C19}} \right) \times [\left( {\upmu{\text{g C19/}}\left( {{\text{response coefficient}} \times {\text{mg sample weight}}} \right)} \right] .$$

The macronutrient and the FA composition of the experimental diets are presented in Table 3.

**Table 3:** Analyzed macronutrient content and fatty acid composition of the experimental diets

Item	N-TAG	E-TAG	E-MDAG
Macronutrient content (wt%)			
Dry matter	90.4	90.4	90.4
Crude protein	24.3	24.0	23.5
Crude fat	7.50	7.50	7.61
Crude fiber	2.72	2.88	2.70
Ash	7.06	7.05	6.84
Gross energy (kcal/kg)	4,244	4,243	4,222
Fatty acid composition (wt%)			
C16:0	36.9	37.1	37.1
C18:0	4.41	4.36	4.73
C18:1n-9	32.6	33.5	31.1
C18:2n-6	22.3	21.6	22.6
C18:3n-3	1.91	1.77	1.96
Minor fatty acids	1.92	1.72	2.56
SFA	42.4	42.3	43.4
MUFA	33.5	34.4	32.0
PUFA	24.2	23.3	24.5

Diets with 6 wt% of native palm oil (N-TAG), re-esterified palm oil (E-TAG), or re-esterified palm oil high in mono- and diacylglycerols (E-MDAG)

All samples were analyzed at least in duplicate

*SFA* saturated fatty acids, *MUFA* monounsaturated fatty acids, *PUFA* polyunsaturated fatty acids

### Controls and Sampling

Feed consumption and weight gain were measured weekly to calculate average daily feed intake, average daily gain, and feed conversion ratio throughout the experiment.

From day 7 to 10, a digestibility balance was carried out using the total-excreta-collection method, according to the European reference method [23]. The last day of the balance, feed consumption was measured and total excreta was collected, weighed and homogenized, and a representative sample was frozen at −20 °C. Contaminants such as feed, feathers, down, and scales were removed. Then, the excreta samples were freeze-dried, ground and kept at 5 °C until further analysis.

Excreta samples were analyzed by the same methods as those described for feeds, to determine the apparent absorption of organic matter, fat (with previous acid hydrolysis, following method 954.02 of the AOAC [17]) , and fatty acids, and to calculate the apparent metabolizable energy of the diets. The apparent absorption of the nutrients (*X*) was calculated as follows:

3 $${\text{Apparent absorption of }}X\,\,(\% ) = \left[ {\left( { \, X{\text{ ingested}} - X{\text{ excreted}}} \right)/X{\text{ ingested}}} \right] \times 100.$$

In the case of the apparent metabolizable energy, the apparent absorption coefficient of gross energy was multiplied by its corresponding feed gross energy.

The postprandial lipemia kinetic study was carried out on the last day of the experiment. After 14 days feeding, chickens were fasted for 5 h, and then re-fed ad libitum for 20 min. Blood samples were drawn by jugular venipuncture at 0, 40, 80, 120, 160 and 200 min following consumption of the experimental diets (one extraction per bird; *n* = 4). Samples were immediately centrifuged at 2,000*g* for 10 min and serum was stored at −20 °C until analysis. Serum TAG concentrations were measured in each sample using a clinical chemistry autoanalyzer (Olympus AU400; Hamburg, Germany) and an enzymatic reagent (glycerol phosphate oxidase, Beckman Coulter; Galway, Ireland).

### Statistical Analysis

Normality of the data and homogeneity of the variance were verified. The effect of diet on FA apparent absorption and growth performance were statistically analyzed by one-way ANOVA with diet as a main factor. Differences between treatment means were tested using Tukey’s correction for multiple comparisons. The cage served as the experimental unit, so there were 6 units per diet.

The effect of diet on postprandial lipemia was statistically analyzed by two-way ANOVA. The model included time of extraction and diet as main factors and the two-way interaction. It was not possible to analyze these data as repeated-measures because each blood extraction was performed in a different animal, due to the limited volume of blood that can be drawn from young chicks. The animal served as the experimental unit, so that there were 4 units per each time-point.

Results in tables are reported as means, differences were considered significant at *P* < 0.05, and trends were discussed at *P* < 0.10. All procedures were carried out using the SAS statistical package (version 9.2, SAS Institute Inc.; Cary, NC).

## Results

### Characterization of Experimental Fats

Because re-esterified oils may have different physicochemical properties than have their corresponding native oil, a detailed characterization of the experimental fats was performed. The chemical analysis of the experimental fats is presented in Table 1. Experimental fats had similar and low levels of MIU. Regarding total FA composition, experimental fats also showed a similar FA profile. This indicated that the esterification of palm acid oil with glycerol did not substantially modify the FA composition of the fat. Therefore, the FA composition of re-esterified palm oils agreed with that reported for native palm oil, being more than 80 wt% of the total FA composed of palmitic (45.0 ± 1.01 wt%) and oleic (38.0 ± 0.94 wt%) acids.

The natural preference for a specific FA positional distribution was evident in N-TAG oil, since FA were not randomly distributed over the three positions of the glycerol molecule. As expected, FA at the *sn*-2 position of N-TAG oil contained less palmitic acid than did those at the *sn*-1,3 positions of the glycerol molecule. On the contrary, although chemical esterification did not result in a complete random distribution of FA, this process raised the fraction of palmitic acid at the *sn*-2 position from 9.63 mol% in N-TAG oil to 17.9 mol% in E-TAG oil, and 14.5 mol% in E-MDAG oil.

Another important difference among experimental fats was their acylglycerol composition. E-TAG oil presented the highest TAG content (86.2 wt%), followed by N-TAG oil (66.6 wt%), and E-MDAG oil (25.6 wt%). In contrast, E-MDAG oil presented the highest DAG content (51.2 wt%), followed by N-TAG oil (19.9 wt%), and E-TAG oil (12.1 wt%). E-MDAG oil also presented an important amount of MAG (23.1 wt%), and N-TAG oil of FFA (11.4 wt%). Because of the relevance in the digestion process, by using ^1^H NMR we found that most FA from MAG and DAG molecules of our experimental fats were located at the *sn*-1,3 positions. Thus, in E-MDAG oil, the isomeric ratio between 1(3)-MAG:2-MAG was 94:6 mol/mol, and that between 1,3-DAG:1(3),2-DAG was 68:32 mol/mol.

The different acylglycerol composition observed in experimental fats was closely related to its glycerol-to-fatty-acid ratio and, in turn, to their subsequent gross energy content. Given that the average heat of combustion of palm FA (9,455 kcal/kg) is more than twice that of glycerol (4,346 kcal/kg), an increase in the glycerol-to-fatty-acid ratio has a negative impact on the gross energy content. N-TAG and E-TAG oils, due to their high TAG content, showed nearly the same glycerol-to-fatty-acid ratio, close to 0.33 mol/mol, (N-TAG oil: 0.34 mol/mol and E-TAG oil: 0.35 mol/mol), and nearly identical gross energy content (N-TAG oil: 9,307 kcal/kg and E-TAG oil: 9,298 kcal/kg). Nevertheless, the glycerol-to-fatty-acid ratio of E-MDAG oil increased up to 0.56 mol/mol, because of its high MAG and DAG content, and this difference represented a decrease of about 4 % on its gross energy content (8,947 kcal/kg).

**Fig. 2:**
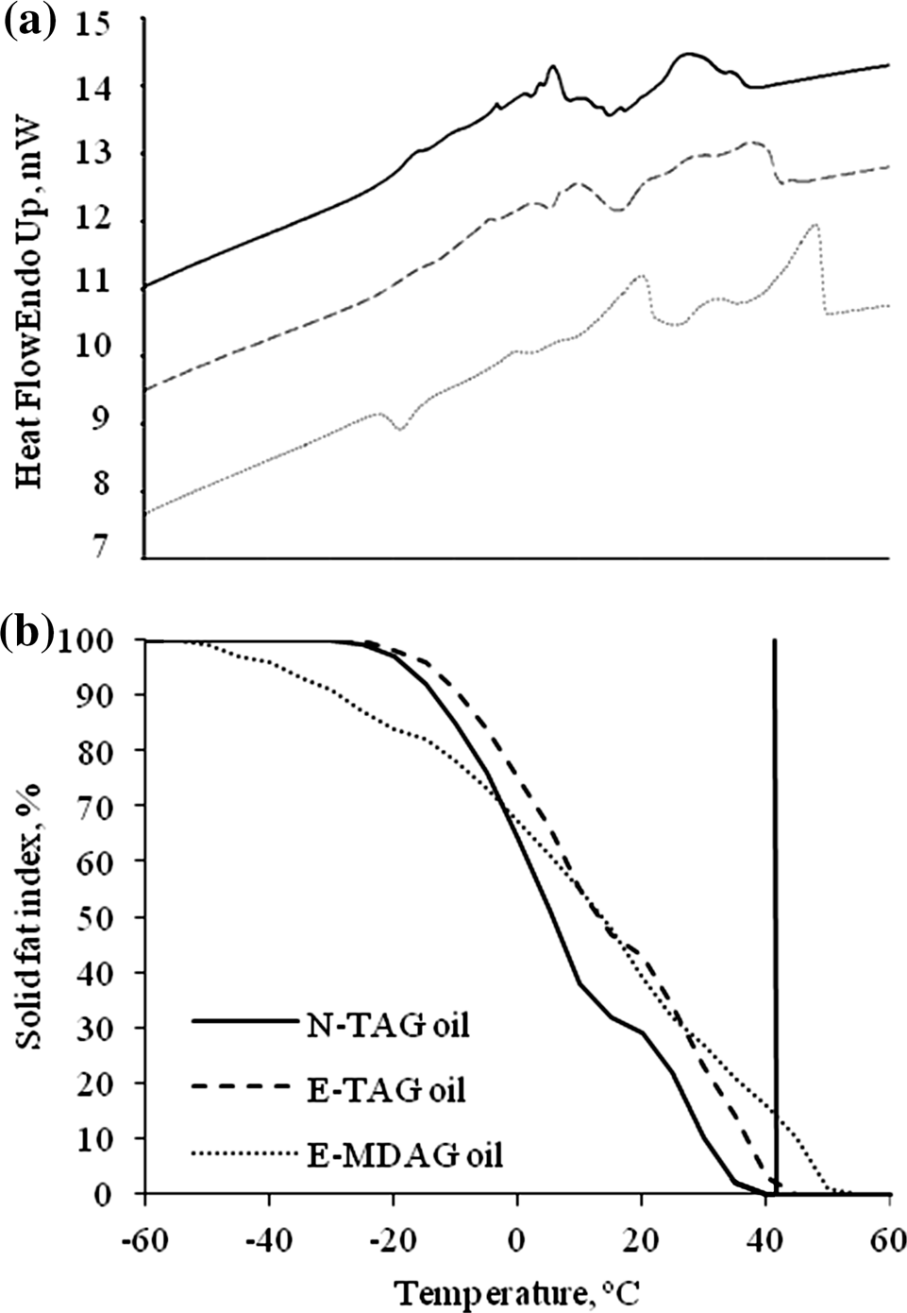
Melting thermogram (**a**) and melting profile (**b**) of native palm oil (N-TAG), re-esterified palm oil (E-TAG), and re-esterified palm oil high in mono- and diacylglycerols (E-MDAG), determined by differential scanning calorimetry. The *vertical line* on Fig. 2b shows the solid fat index at chicken’s body temperature (41.5 °C)

The melting thermograms (Fig. 2a), determined by DSC, allowed us to calculate the total and partial melting enthalpies. Although experimental fats showed different and highly complex melting patterns, a similar amount of energy was needed to bring them from the solid to the liquid state (N-TAG oil: 106, E-TAG oil: 99, and E-MDAG: 104 J/g). The melting profile of these oils (Fig. 2b), obtained by calculations of the above enthalpies, showed that N-TAG and E-TAG oils had nearly the same melting profile and the same melting range, although N-TAG oil started to melt and finished melting earlier than did E-TAG oil (N-TAG oil: from –25 to 35 °C and E-TAG oil: from –20 to 40 °C). However, E-MDAG oil started to melt earlier and finished melting later, expanding its melting range (from −50 to 50 °C). Nevertheless, the most important physical property of fats in lipid nutrition is their solid fat index at the chicken’s body temperature (41.5 °C). While N-TAG and E-TAG oils were totally or almost completely liquid at 41.5 °C (N-TAG oil: 0 wt% and E-TAG oil: 1 wt%), E-MDAG oil still had a 16 wt% of solid fat index at this temperature.

### Digestibility Balance

The effects of dietary treatments on the apparent absorption coefficients are presented in Table 4. As expected, unsaturated FA were better absorbed than were SFA, and stearic acid was less absorbed than was palmitic acid. However, no statistically significant differences were observed among treatments for fat and FA apparent absorption (*P* > 0.05), although E treatments, contrary to what was expected, showed numerically lower apparent absorption coefficients than did N-TAG. In this sense, neither statistical differences among treatments were observed for apparent metabolizable energy, which was consistent with the organic matter apparent absorption results (*P* > 0.05). The lower gross energy found for E-MDAG oil was too small to be reflected in the feed, since fat only accounted for 17 % of the total-feed gross energy.

**Table 4:** Apparent absorption coefficients (%) according to different fat sources in diet

Item	Dietary treatments^a^			RMSE^b^	*P* values
	N-TAG	E-TAG	E-MDAG		
AME (kcal/kg)	2,976	2,888	2,883	119.0	0.34
Organic matter	69.8	68.3	69.0	2.01	0.63
Crude fat	52.5	43.7	39.2	13.48	0.25
Total fatty acids	53.8	47.9	38.9	12.61	0.16
SFA	44.9	39.0	31.5	11.89	0.18
MUFA	61.5	54.9	43.8	13.97	0.12
PUFA	58.6	53.6	45.8	12.55	0.23
C16:0	46.6	40.8	32.7	11.64	0.15
C18:0	31.9	25.3	17.1	13.08	0.18
C18:1n-9	62.2	55.7	45.3	13.61	0.13
C18:2n-6	58.3	53.2	45.2	12.64	0.22

*AME* apparent metabolizable energy, *SFA* saturated fatty acids, *MUFA* monounsaturated fatty acids, *PUFA* polyunsaturated fatty acids

^a^Diets with 6 wt% of native palm oil (N-TAG), re-esterified palm oil (E-TAG), or re-esterified palm oil high in mono- and diacyglycerols (E-MDAG)

^b^ *RMSE* Root mean square error of six observations per treatment (the experimental unit is the cage)

### Postprandial Lipemia

**Fig. 3:**
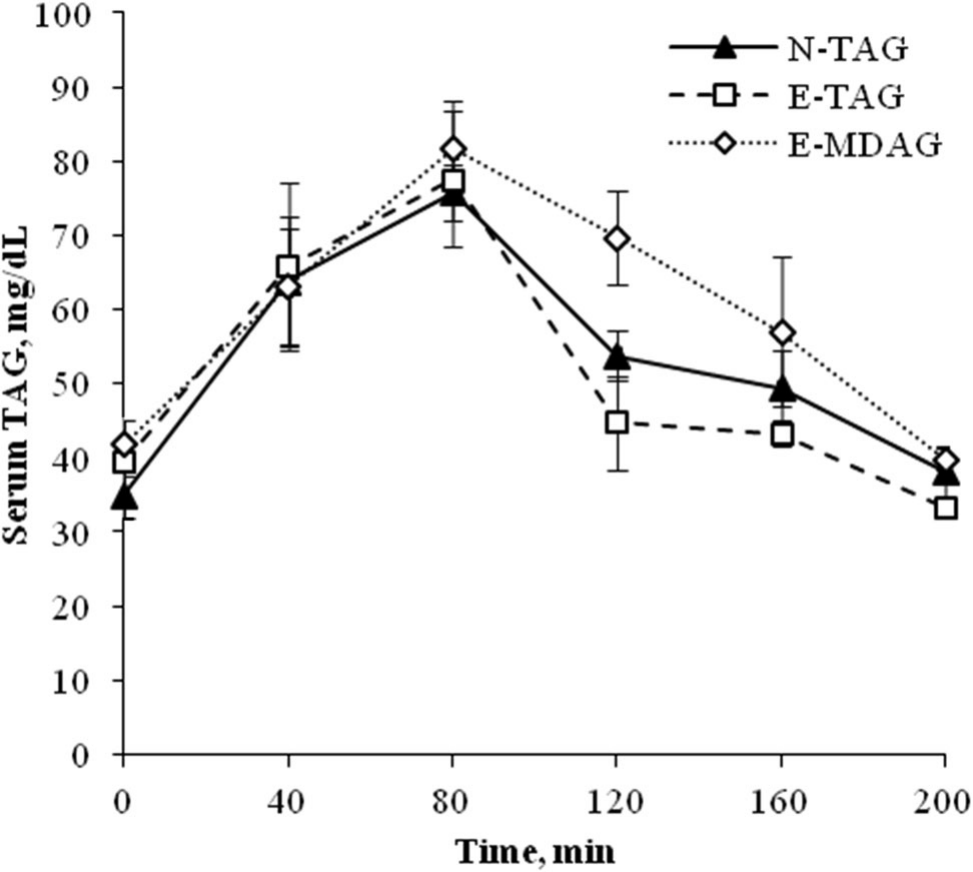
Postprandial triacylglycerol (TAG) concentrations in serum of broiler chicks, following consumption of native palm oil (N-TAG), re-esterified palm oil (E-TAG) or re-esterified palm oil high in mono- and diacylglycerols (E-MDAG) supplemented diets. Values are expressed as means ± SE, *n* = 4 for each time-point

The fasting levels of serum TAG (0 min) did not differ significantly between treatments (N-TAG: 35.0 ± 3.19 mg/dL, E-TAG: 39.5 ± 2.02 mg/dL, and E-MDAG: 41.8 ± 3.22 mg/dL; *P* = 0.29). Postprandial TAG concentrations after feeding the experimental diets are shown in Fig. 3. Statistical analysis showed that serum TAG concentrations differed significantly (*P* < 0.001) throughout the postprandial period. However, there were no significant differences (*P* = 0.07) in the postprandial responses among treatments, although E-TAG tended to show lower serum TAG concentrations when compared with E-MDAG (*P* = 0.07).

Following consumption of experimental diets, the rates of lipid transport sharply increased. The serum TAG concentration rose to a postprandial level of about 78.3 ± 3.68 mg/dL at 80 min after feeding. Then, serum TAG concentrations rapidly returned to baseline levels, with no statistically significant differences among treatments.

### Growth Performance

The effect of dietary fat source on growth-performance traits is reported in Table 5. Differences were only found for the average daily feed intake. The increased MAG and DAG content of E-MDAG treatment caused a statistically (*P* = 0.01) or a nearly statistically (*P* = 0.10) significant decrease in feed intake, when compared with E-TAG and N-TAG treatments, respectively. Weight gain, feed conversion, and final body-weight were not statistically affected by the diet (*P* > 0.05).

**Table 5:** Growth performance of broiler chickens according to different fat sources in diet

Item	Dietary treatments^a^			RMSE^b^	*P* values
	N-TAG	E-TAG	E-MDAG		
ADFI (g/day per bird)	32.9^a, b^	33.8^a^	30.8^b^	1.59	0.02
ADG (g/day per bird)	24.1	23.8	22.1	1.79	0.14
FCR (g/g)	1.37	1.42	1.40	0.064	0.42
BW at 13 days (g)	359	357	337	25.4	0.26

Values within the same row with no common superscript are significantly different, *P* < 0.05

*ADFI* average daily feed intake, *ADG* average daily gain, *FCR* feed conversion ratio, *BW* body weight

^a^Diets with 6 wt% of native palm oil (N-TAG), re-esterified palm oil (E-TAG), or re-esterified palm oil high in mono- and diacylglycerols (E-MDAG)

^b^ *RMSE* Root mean square error of six observations per treatment (the experimental unit is the cage)

## Discussion

### Fatty Acid Positional Distribution Within Acylglycerol Molecules

Our hypothesis was that the chemical esterification of palm acid oil with glycerol would increase SFA content at the *sn*-2 position, and therefore its absorbability, in particular that of palmitic acid. Although E-TAG oil showed a higher percentage of palmitic acid at the *sn*-2 position than did N-TAG oil, no improvements in the apparent absorption of this FA or other individual FA were observed in this study. Similarly, Smink et al. [14] also found that randomization of palm native oil did not significantly affect fat and FA absorption in young broiler chickens. In contrast, other studies carried out with human newborn infants [24], rats [25–27] and broiler chickens [28, 29] reported that palmitic acid present at the *sn*-2 position of dietary TAG was more readily absorbed than was palmitic acid at the *sn*-1,3 positions. While most of these studies used fats with a high palmitic acid content at the *sn*-2 position (from 33 to 84 mol%), in our study E-TAG oil only had 17.9 mol% of the total palmitic acid content located at the *sn*-2 position.

Once in the blood, SFA in the *sn*-2 position may delay the TAG clearance. The increased proportion of high melting MAG may alter the physical properties of the surface layer of remnant particles, impeding further hydrolysis, and slowing removal by the liver [30, 31]. However, in our experiment, E-TAG animals did not show a more prolonged postprandial lipemia response than did N-TAG animals. Several authors [32–35] did not find significant differences in plasma lipids of adult men and women after consuming TAG with different FA positional distribution. Because the extent of postprandial lipemia is determined by both rates of absorption and clearance of dietary TAG, the reasons for not finding differences in the peak TAG concentration or the time to maximal TAG concentration following consumption of dietary treatments suggests that fats studied were digested and cleared from the blood at the same rates, or that the small difference observed in the FA positional distribution of our experimental fats, added to the high individual variations, prevented us from seeing any statistical effect on the postprandial lipemia response.

Regarding growth performance traits, the lack of differences found between N-TAG and E-TAG groups are in good agreement with the findings observed by Lin and Chiang [28] and Smink et al. [14], who also fed broiler chickens with native and randomized palm oils.

### Acylglycerol Composition

For the second objective, the hypothesis tested was that the presence of MAG and DAG molecules, due to their amphiphilic properties, would act as emulsifying agents, able to enhance fat digestion and absorption. In the study of Garrett and Young [5], the efficacy of free oleic acid in enhancing palmitic acid absorption in broiler chickens was compared with that of the MAG of oleic acid (mono-olein). Mono-olein, due to its higher micellar solubility, was superior to oleic acid in promoting the absorption of palmitic acid at several different ratios. Nevertheless, in our study, although the increased glycerol-to-FA ratio of E-MDAG oil resulted in a greater amount of MAG and DAG molecules, no improvements in the apparent absorption of fat and individual FA were observed in comparison with N-TAG. Taguchi et al. [36] reported that the fecal excretion of FA after feeding rats with DAG was almost the same as that with TAG, suggesting that the intestinal absorption of DAG was comparable to that of TAG, although they used fat sources rich in oleic and linoleic acids.

The reason why MAG and DAG molecules did not enhance micellar solubilization may be due to their increased melting point. Each acylglycerol molecule has specific melting and crystallization characteristics. It is well known that tri-saturated TAG (PPP with a melting point of 66.4 °C, where P is palmitic acid), due to their high melting temperatures, are almost not digested [6]. However, di-saturated DAG have even higher melting points (74.9 and 70.1 °C for 1,3-PP and 1(3),2-PP, respectively), and saturated MAG are still crystalline above body temperature (1(3)-P and 2-P have melting points of 70.5 and 68.5 °C, respectively) [6]. Thus, the presence of high amounts of saturated MAG and DAG, may have promoted the formation of crystalline structures on the surface of fat globules, preventing the hydrolytic action of pancreatic lipase. In addition, given that the melting points of saturated 1,3-DAG and 1(3)-MAG, major isomers in our experimental fats, are higher than are their respective 1,2-DAG and 2-MAG isomers, may have contributed to further increase the solid fat index at the chicken’s body temperature of E-MDAG oil (16 wt%).

Otherwise, Kondo et al. [37] and Murata et al. [38] reported that the main end-products of lipase action on 1,3-DAG and 1(3)-MAG were free glycerol and FFA. It is not surprising, therefore, that the increased MAG and DAG content of E-MDAG, rather than benefitting, may have impaired the absorption of fats.

There is still one more possible hypothesis. It has been suggested that the end-products of 1(3)-MAG and 1,3-DAG digestion are less readily re-synthesized to TAG inside enterocytes [37, 38], because the lack of 2-MAG forces the TAG re-synthesis to proceed via the glycerol-3-phosphate pathway, which is less active than the 2-MAG pathway. As a result, this could lead to a slower entry of the products of fat digestion inside enterocytes, which in turn may have caused changes in lipid metabolism. Compared with consumption of TAG, several studies have reported that consumption of DAG produces lower postprandial [38–41] and fasting serum TAG concentrations [42, 43] in humans, rats and pigs, which has also been related to a higher FA β-oxidation activity in the small intestine [44] and in the liver [45]. However, there are several reasons related to our experimental design that can explain why we did not observe changes on postprandial lipemia response among treatments. Most studies assessed postprandial lipemia after only one single meal; however, in our study, the animals had been fed with experimental fats since the first day of life. In addition, while previous studies used TAG and DAG oils with oleic and linoleic acids as the major FA, our experimental fats were rich in palmitic and oleic acids. Other limitations of our study may have been the small amount of fat consumed by the birds and the inability to perform serial blood extractions from each animal.

Concerning growth performance, the significantly lower average daily feed intake observed in the E-MDAG group could be in good agreement with the reported lower feeling of hunger and appetite observed in women fed a DAG oil-rich diet [46]. Authors suggested that this could be related to the higher β-hydroxybutyrate concentrations found in blood, as a result of the increased fat oxidation suggested above, which are inversely associated with appetite and food intake [47]. However, other studies where animals were fed TAG and DAG oil-rich diets did not find differences in either feed intake or weight gain [36, 45].

Taken together, it is concluded that the chemical esterification of palm acid oil with glycerol can be used to obtain re-esterified palm oils with the same degree of saturation and isomeric state of FA, but with a different intramolecular structure and melting profile than native palm oil. Despite the differences observed for the FA positional distribution within acylglycerol molecules and acylglycerol composition in re-esterified palm oils, these fats did not alter fat absorption, postprandial lipemia or growth performance, compared to native palm oil, so they can be used as alternative fat sources in broiler chick diets.

## Abbreviations

DAG: Diacylglycerol(s)
DSC: Differential scanning calorimetry
FA: Fatty acid(s)
FFA: Free fatty acid(s)
MAG: Monoacylglycerol(s)
MIU: Moisture, impurities and unsaponifiable matter
MUFA: Monounsaturated fatty acid(s)
NMR: Nuclear magnetic resonance
PUFA: Polyunsaturated fatty acid(s)
SFA: Saturated fatty acid(s)
TAG: Triacylglycerol(s)
